# Simplified approaches for the development of an ELISA to detect circulating autoantibodies to p53 in cancer patients

**DOI:** 10.1186/1472-6750-8-16

**Published:** 2008-02-20

**Authors:** Ratchada Cressey, Saranya Pimpa, Busyamas Chewaskulyong, Nirush Lertprasertsuke, Somchareon Saeteng, Chatchai Tayapiwatana, Watchara Kasinrerk

**Affiliations:** 1 Division of Clinical Chemistry, Faculty of Associated Medical Sciences, Chiang Mai University, Chiang Mai 50200, Thailand; 2 Department of Internal Medicine, Faculty of Medicine, Chiang Mai University, Chiang Mai 50200, Thailand; 3 Department of Pathology, Faculty of Medicine, Chiang Mai University, Chiang Mai 50200, Thailand; 4 Department of Surgery, Faculty of Medicine, Chiang Mai University, Chiang Mai 50200, Thailand; 5 Division of Clinical Immunology, Faculty of Associated Medical Sciences, Chiang Mai University, Chiang Mai, 50200, Thailand; 6 Biomedical Technology Research Unit, National Center for Genetic Engineering and Biotechnology, National Science and Technology Development Agency, Thailand

## Abstract

**Background:**

The recognition that human tumors stimulate the production of autoantibodies has initiated the use of this immune response as serological markers for the early diagnosis and management of cancer. The enzyme-linked immunosorbent assay (ELISA) is the most common method used in detecting autoantibodies, which involves coating the microtiter plate with the tumor associated antigen (TAA) of interest and allowing serum antibodies to bind. The patient's sample is directly in contact with the coating antigen so the protein used for coating must be pure to avoid non-specific binding. In this study, a simplified method to selectively and specifically immobilize TAAs onto microtiter plates in order to detect circulating autoantibodies in cancer patients without prior purification process was described. Wild type full-length p53 protein was produced in fusion with biotin carboxyl carrier peptide (BCCP) or hexahistidine [(His)6] using pAK400 and pET15b(+) vectors, respectively. The recombinant p53 fusion protein produced was then subjected to react with either a commercial p53 monoclonal antibody (mAb) or sera from lung cancer patients and healthy volunteers in an enzyme-linked immunosorbent assay (ELISA) format.

**Results:**

Both of the immobilized p53 fusion proteins as well as the purified (His)6-p53 fusion protein had a similar dose response of detection to a commercial p53 mAb (DO7). When the biotinylated p53-BCCP fusion protein was used as an antigen to detect p53 autoantibodies in clinical samples, the result showed that human serum reacted strongly to avidin-coated microwell even in the absence of the biotinylated p53-BCCP fusion protein, thus compromised its ability to differentiate weakly positive sera from those that were negative. In contrast, the (His)6-p53 protein immobilized directly onto Ni+ coated microplate was able to identify the p53 autoantibody positive serum. In addition, its reactivity to clinical serum samples highly correlated with those obtained from using purified p53 as an antigen (R = 0.9803, p < 0.0001). Moreover, this directly immobilized p53 antigen can clearly differentiate p53 autoantibody positive sera in cancer patients from healthy volunteers' sera.

**Conclusion:**

A method of coating directly and specifically TAAs onto a microtiter plate without the purification processes was developed in this study. Although in this study only one tumor antigen was examined, the simplicity and the ability of coated antigens to identify p53 specific autoantibodies in serum accurately might enable a larger panel of TAAs specific autoantibodies to be explored as serological markers for cancer.

## Background

The use of recombinant proteins has increased greatly in recent years and as a consequent the development of techniques for their purification has significantly increased. The advantage of using fusion proteins to facilitate purification and detection of the recombinant protein is now widely recognized. More than 20 years ago it was discovered that many natural proteins have metal binding sites that can be utilized for protein purification. An amino acid sequence consisting of 6 or more consecutive histidine (His) residues can act as a metal binding site. If a target protein is produced in fusion with a His-tag sequence, it can be purified using a solid support that is covalently modified to displays a heavy metal ion like Ni^2+ ^or Zn^2+ ^on the surface. Immobilized metal affinity chromatography (IMAC) has been the most common technique used for protein purification and a His-tag sequence can be placed on either the N-terminal or C-terminal of a target protein by using commercially available vectors. Recently, the use IMAC for protein purification has expanded due to the development of improved chelating agents that permit high-affinity coordination of metal ion by both the immobilized chelation agents and the protein [1]. Resins coupled with nitriloacetic acid (NTA) are the most suitable solid support using metal ions with a coordination number of six, such as Ni^2+^, because quadridentate NTA occupies four coordination positions, leaving two positions available for tight, but reversible, interactions with target proteins [2].

The biotin-avidin/strepavidin system is used in numerous biotechnological and diagnostic applications, primarily due to the high affinity of the proteins avidin and strepavidin to the small biotin molecule [3]. A small biotin tag has frequently been used for detection as well as for the purification of proteins [4]. This tag can serve as an anchor for immobilization of proteins onto solid surfaces. Surfaces coated with avidin or strepavidin that efficiently bind biotinylated molecules are readily available, as are chemical reagent for biotinylation of certain functional group. However, the disadvantages of chemical biotinylation are that it often results in the inactivation of the protein and may yield heterogenous reaction product unsuitable for structural studies. It has been demonstrated that some natural protein are post-translationally biotinylated at a unique lysine residues by the catalysation of biotin protein ligase [5,6]. In *Escherichia coli*, (*E.coli*), there is only one post-translationally biotinylated protein, namely, the biotin carboxyl carrier protein (BCCP) [7]. Thus, when this domain is fused to a recombinant protein, it will be post-translationally biotinylated *in vivo *by the endogenous biotin ligase of *E.coli *[8].

It is well recognized that cancer can initiate autoimmunity [9]. Circulating antibodies to autologous tumour cell antigens in cancer patients have been reported in several studies [10,11]-[12]. Although factors leading to the production of such autoantibodies are not completely understood, available data suggested that many of the target antigens are cellular proteins whose aberrant expression can lead to tumourigenesis such as *HER-2/neu*, *ras*, *c-myc*, *survivin *and *p53 *[9,13]. Among these the most extensively studies cancer-associated antigens is p53, a tumour suppressor protein. Autoantibodies to p53 in cancer was first reported in 1982 [14] and since then there have been over 100 reports confirming and extending this finding [15]. As there is generally absence of these antibodies in normal individuals and non-cancer conditions, there has been interested in using these autoantibodies as serological markers for cancer diagnosis. Herein we described a simplified method to selectively immobilize cancer-associated recombinant antigens onto microtiter plates in order to detect circulating autoantibodies in cancer patients without prior purification process.

## Results

### Expression and purification of p53 fusion protein

**Figure 1:**
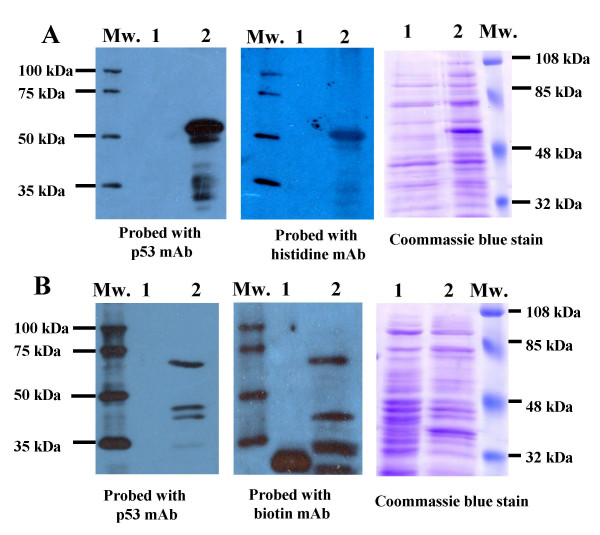
**Western blot analysis of (His)6-p53 fusion protein (A) and biotinylated p53-BCCP fusion protein (B) expressed in *E. coli *BL21(DE3)pLysS strain and Origami strain, respectively**. Cell lysate from *E. coli *BL21(DE3)pLysS strain transformed with pET15b empty vector was also analyzed as a negative control for analysis of (His)6-p53 fusion protein. *E.coli *Origami strain transformed with pAK400 harbouring CD147 encoding DNA was analysed as a negative control for analysis of biotinylated p53-BCCP fusion protein. Fifteen micrograms of crude cell lysates were electrophoresed and electroblotted onto PVDF membranes. The PVDF membrane was incubated with either mouse anti-p53 mAb (DO7), mouse anti-histidine mAb or mouse anti-biotin mAb, respectively. Lane 1, bacterial cell lysate from negative controls; lane 2, lysate from p53 expressing cells

After obtaining and confirming DNA sequences of expression constructs, the established bacterial clones were cultured and activated with IPTG in order to produce recombinant p53 protein antigen. Western blot analysis revealed that the (His)6-p53 fusion protein reacted with both anti-p53mAb and anti-histidine mAb producing a band at molecular weight of 53 kDa (Figure 1A). BCCP in fusion with p53 mimics the natural substrate for *E. coli *biotin ligase, thus the p53-BCCP fusion protein is readily biotinylated *in vivo*. The p53-BCCP fusion protein reacted with anti-p53mAb producing an expected size of 67 kDa (BCCP protein is 14 kDa in size); however, this antibody also gave a reactive band at molecular weight around 48 kDa, which is suspected to be a small un-tagged p53 protein as this band was not recognized by anti-biotin mAb (Figure 1B). In addition, anti-biotin mAb appeared to react with a number of smaller bands less than 48 kDa.

**Figure 2:**
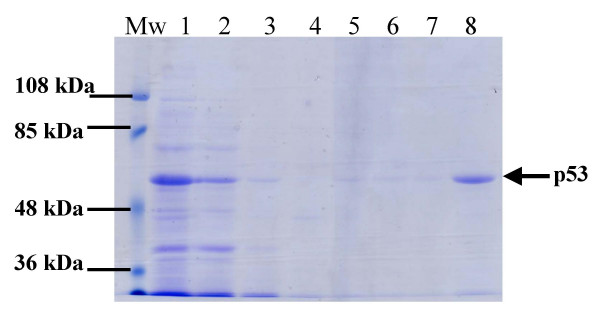
**Coomassie blue staining showing the purification process of (His)6-p53 fusion protein using nickel chelated affinity chromatography**. 15 μl of each fraction were resolved through 10% SDS-PAGE, followed by Coomassie blue staining. The molecular weight of the purified protein was ~53 kDa. M, molecular weight marker; lane 1, bacterial cell lysate containing (His)6-p53 fusion protein; lane 2, flow-through fraction of the nickel chelated affinity chromatography; lane 3, washed fraction with binding buffer, lane 4–7, washed fractions with washing buffer containing increasing concentration of imidazole (20, 60, 40 and 80 mM, respectively), lane 8, the purified (His)6-p53 fusion protein obtained after eluting with 1 M imidazole

The (His)6-p53 fusion protein was purified by using metal-affinity chromatography. As shown in Figure 2, the fusion protein was purified to at least 90% purity when analyzed by SDS-PAGE. These results indicated that the purified full-length p53 protein could be used as antigen for the detection of p53 autoantibody to monitor the immune response against p53 in patients with cancer.

### Immobilized p53 recombinant protein directly from crude lysate onto microtiter plates shows a dose response of detection with a commercial antibody

**Figure 3:**
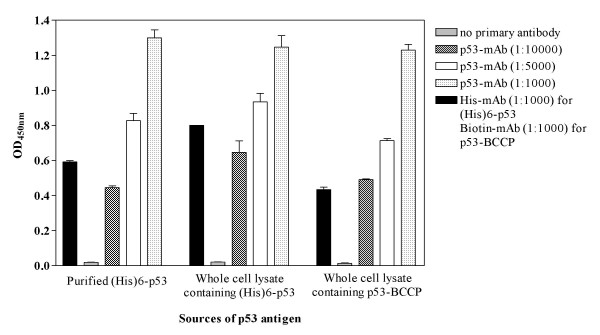
**ELISA experiment showing reactivity of three different sources of p53 antigen to various commercial antibodies**. The purified (His)6-p53 fusion protein 1 μg/ml (A) and (His)6-p53 fusion protein 100 μg/ml directly immobilized onto nickel coated microtiter plate (B) were subjected to react with various amounts of anti-p53 mAb (0, 1:10000, 1:5000 and 1:1000 dilution) and anti-histidine mAb (1:1000 dilution). The p53-BCCP fusion protein directly immobilized onto avidin-coated microtiter plate were reacted with various amounts of anti-p53 mAb (0, 1:10,000, 1:5,000 and 1:1,000 dilution) and anti-biotin mAb (1:1000 dilution). Experiment was performed in triplicate and error bars represent standard deviation.

Wild type full-length p53 protein was produced in fusion with BCCP or  hexahistidine-tag (His)6. BCCP mimics the natural substrate for E. coli  biotin ligase, thus the p53-BCCP fusion protein produced from this  expression system is readily biotinylated in vivo and without requiring  any further purification step can be selectively immobilized onto avidin  coated microtiter plates (Figure 3). In order to selectively immobilize (His)6-p53 fusion protein directly from crude cell lysate, the NTA derivative (N,N-bis [carboxymethy]lysine) was coupled to a microtiter plate and charged with Ni^2+ ^ions. Crude lysate extracted from bacteria containing (His)6-p53 fusion protein was applied directly onto nickle coated microplate along with a purified (His)6-p53 fusion protein. The directly immobilized (His)6-p53 proteins as well as the purified (His)6-p53 fusion protein had a similar reactivity to anti-histidine mAb and a similar dose response to anti-p53 mAb (Figure 3).

### Reactivity of sera from lung cancer patients to an immobilized p53 antigen

Although the immobilized p53 antigens reacted with a commercial p53 mAbs in a dose response manner, human serum is far more complex than the commercial antibody. Therefore, we tested whether the immobilized p53 antigens could be used to detect p53 autoantibodies in clinical samples. In order to identify p53 autoantibody producing sera, the purified (His)6 fusion p53 was resolved through SDS-PAGE, transferred onto a solid support PVDF membrane after which it was cut into small strips and probed with serum from each cancer patient, individually.

**Figure 4:**
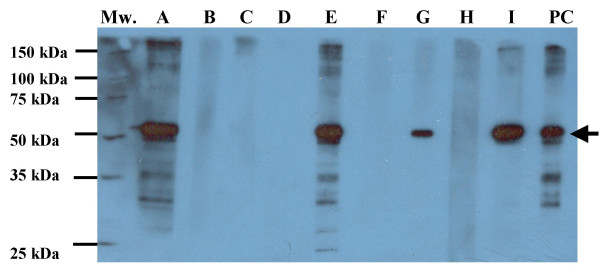
**Representative results of serum p53 autoantibodies of lung cancer patients detected by immunoblotting**. PVDF membrane containing 1 μg/strip of the purified (His)6-p53 fusion protein was incubated with 1:5,000 p53 mAb DO7 as positive control, 1:200 diluted sera from lung cancer patients, respectively. Lane PC, positive control: probing with p53 mAb DO7, lane A-I, probing with serum from lung cancer patients.

Figure 4 shows representative western blot result from 9 cancer patients, the last strip was probed with a commercial p53 mAb, which was used as a positive control (PC). Patients whose sera showed reactive bands at the same size as the positive control were those believed to contain p53 autoantibodies. Two negative sera, Strip D (no background signal, referred as negative 1) and Strip B (with weak non-specific reactivity, referred as negative 2) and 2 positive sera, Strip G (weakly positive, referred as positive 1) and Strip E (strongly positive, referred as positive 2) were chosen and subjected to ELISA.

**Figure 5:**
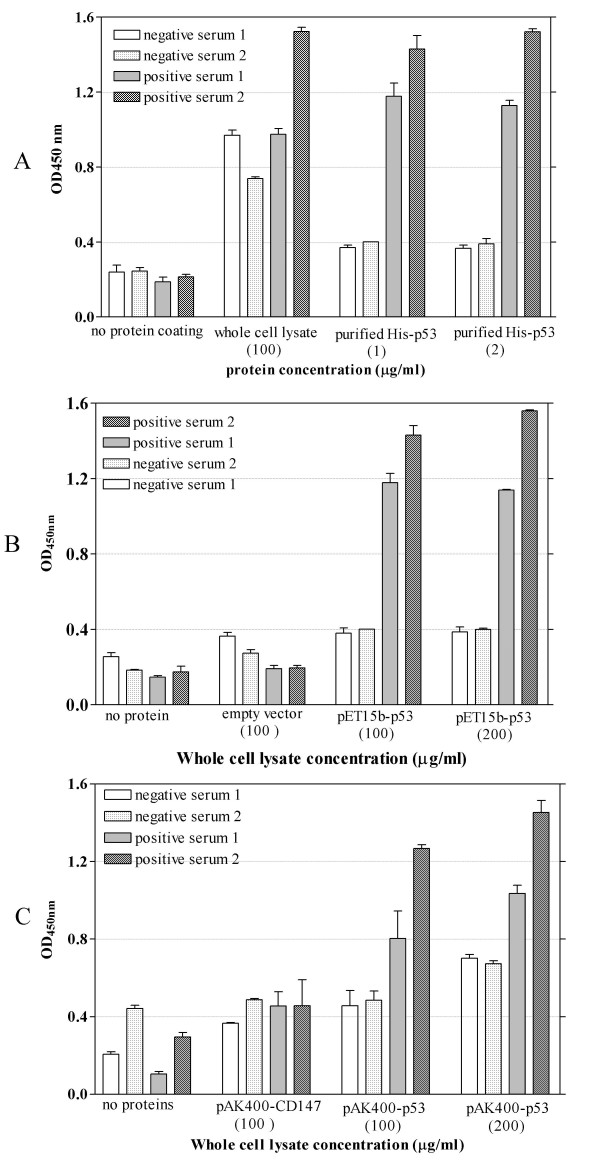
**ELISA experiment with sera from lung cancer patients**. Two negative sera and two positive sera for producing p53 autoantibody proven by Western blot analysis (as shown in Figure 4) at 1:200 dilution were subjected to react with the purified (His)6-p53 recombinant proteins at concentration of 0, 1 and 3 μg/ml immobilized onto un-modified microplate along with crude lysate containing (His)6-p53 fusion protein (A), or crude lysate containing (His)6-p53 recombinant proteins directly immobilized onto nickel coated microplate at concentration of 0, 100, 200 μg/ml along with negative cell lysate control from pET15b(+) empty vector transformed cells (B), and crude lysate containing biotinylated p53-BCCP fusion proteins immobilized onto avidin-coated microplate at concentration of 0, 100, 200 μg/ml along with biotinylated CD147-BCCP containing cell lysate as a negative control (C) Experiment was done in triplicate and error bar represent standard deviation.

To test whether the immobilized p53 antigens could detect p53 autoantibodies in clinical serum samples, different concentrations (100 and 200 μg protein/ml) of crude cell lysate containing (His)6-p53 protein or p53-BCCP protein along with their respective negative controls were applied onto Ni^2+^-coated or avidin coated microplate respectively and reacted with patient's sera known to be positive or negative for p53 autoantibodies. This ELISA experiment was performed in comparison with those utilizing the purified (His)6-p53 as an antigen. Figure 5A shows that the purified (His)6-p53 protein antigen at each concentrations was able to clearly differentiate p53 autoantibody positive sera from those that were negative. In contrast, the crude cell lysate containing (His)6-p53 antigen directly immobilized onto an un-modified microplate failed to do so. Sera from cancer patients were also subjected to react with (His)6-p53 proteins immobilized directly onto Ni+ coated microplate. As shown in Figure 5B, both concentrations of the immobilized p53 antigen could differentiate between p53 Ab positive and negative sera; however, the lower concentration of 100 μg/ml was chosen for further investigations.

When the biotinylated p53-BCCP fusion protein was used as an antigen and subjected to react with patient's sera. The result showed that human serum reacted strongly to the antigen free avidin-coated microwell and the irrelevant biotinylated protein (CD147)-coated microwell (Figure 5C). The reactivity was significantly increased in response to the amount of biotinylated p53-BCCP contained bacterial cell lysate coated; however, the fold difference between positive sera and the negative sera were less when biotinylated p53-BCCP antigen was used in comparison to (His)6-p53 proteins.

### Reactivity of sera from cancer patients to the (His)6-p53 protein directly immobilized from crude lysate correlates with reactivity to the purified (His)6-p53 antigen

To test reproducibility of the assay, control serum was prepared by combining all p53 autoantibody positive sera together; the pooled serum was then divided into small aliquots and kept at -70°C until use. Control serum was tested twenty times on one plate to assess within-assay CV, which was found to be 6.2%. This control serum was later subjected to ELISA along with clinical samples, the result from separate assays showed the inter-assay precision was 10%.

**Figure 6:**
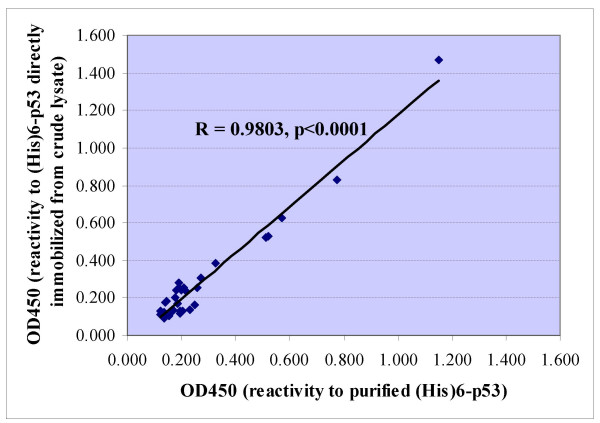
Reactivity of sera from 30 lung cancer patients to purified (His)6-p53 fusion proteins at concentration of 1 μg/ml (x axis) and (His)6-p53 fusion proteins at concentration of crude lysate at 100 μg/ml directly immobilized onto nickel coated plate (y axis).

To test whether the (His)6-p53 protein directly immobilized from crude lysate could replace the purified (His)6-p53 protein to be used as an antigen to detect p53 autoantibodies in clinical samples, 30 sera from lung cancer patients were detected for p53 autoantibody by ELISA using the two different prepared antigens. In Figure 6, a significant correlation of the reactivity from patient's sera to the two antigens was demonstrated (R = 0.9803, p < 0.0001). Although (His)6-p53 antigens directly immobilized from crude lysate showed a slightly higher OD_450 nm _with patient's sera (mean = 0.289, SD = 0.281) than those using purified (His)6-p53 (mean = 0.277, SD = 0.224), it was not statistically different (paired t-test, p = 0.390).

### Reactivity of sera from lung cancer patients and healthy volunteers to a directly immobilized p53 antigen

**Figure 7:**
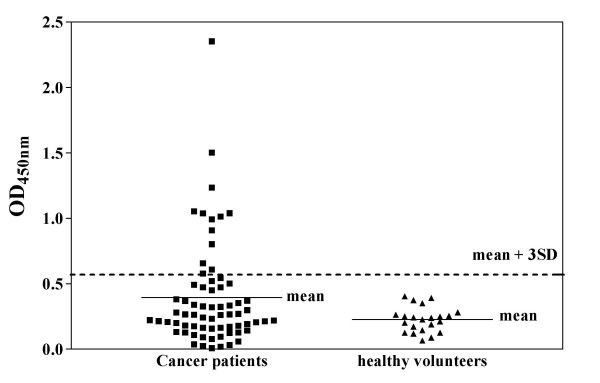
Reactivity of sera from healthy volunteers in comparison to sera from lung cancer patients to (His)6-p53 fusion proteins at concentration of crude lysate at 100 μg/ml directly immobilized onto nickel coated plate.

Since there is a significant correlation of the reactivity of sera from cancer patients to the directly immobilized antigens from crude lysate to the purified 6(His)-p53 antigen, we further tested whether sera from healthy volunteers would show different reactivity to this directly immobilized antigen in comparison to sera from cancer patients. The results are shown in Figure 7.

The average OD450 nm from 22 non-smoker healthy volunteers with no known diagnosis of cancer was 0.227 (SD = 0.0954). The cut off value was calculated by combining the mean OD_450 nm _of sera from healthy volunteers and 3SD, which was found to be around 0.523. Using this cut-off value, 12 of 68 (17.6%) sera from cancer patients (7 with adenocarcinoma, 5 with squamous cell carcinoma) possessed an OD_450 nm _above this value. Western blot analysis performed on sera from cancer patients with OD_450 nm _above 0.523 and sera from healthy volunteers showed that only sera from cancer patients with OD_450 nm _above 0.800 (9 (5 with adenocarcinoma, 4 with squamous cell carcinoma) out of 12 cancer patient's sera examined) gave reactive bands at 53 kDa (data not shown). Nevertheless, a larger group of samples needs to be investigated in order to decide a definitive cut-off value.

## Discussion

The enzyme-linked immunosorbent assays are the most commonly used antibody detection method. This method involves coating the detection plate with the antigen of interest and allowing serum antibodies to bind to protein for later labeling and detection. The patient serum is in direct contact with the antigen so the protein used for coating must be sufficiently pure to avoid non-specific binding. For example, highly purified p53 recombinant protein is necessary for such an assay. In this study, a simplified method to selectively and specifically immobilize tumour-associated recombinant antigens onto microtiter plates in order to detect circulating autoantibodies in cancer patients without prior purification process was described. Wild type full-length p53 protein was produced in fusion with BCCP or hexahistidine-tag (His)6. The BCCP serves as an *in vivo *substrate mimic for *E. coli *biotin ligase, thus p53-BCCP fusion protein produced from this expression system is readily biotinylated *in vivo *and without requiring any further purification step can be selectively immobilized onto avidin coated microtiter plate. In order to selectively immobilize (His)6-p53 fusion protein, the NTA derivative (N,N-bis [carboxymethy]lysine) was couple to an ordinary carboxylated polystyrene microtiter plate and charged with Ni^2+ ^ions. Although both of the directly immobilized p53 antigens from crude lysate bind to the commercial p53 monoclonal antibody in a dose dependent manner, strong background was observed when the biotinylated p53-BCCP fusion protein was immobilized and reacted with patient's sera. One possible reason for explaining why human sera react strongly to antigen free avidin-coated plates or the irrelevant biotinylated protein antigen is that humans may have naturally developed antibodies against avidin and/or biotin. Indeed, it has been previously reported that human sera contain natural antibodies to the egg-white glycoprotein avidin [16]. Of 270 samples tested, all contained antibodies to different extents, mainly IgG and IgM classes, and were capable of activating the complement system. Another problem that we encountered producing biotinylated p53-BCCP was that a fraction of untag-p53 protein product was obtained. This stilled happened, although to a lesser extent, even after a careful optimization of the culture conditions. In addition, there appeared to be a number of smaller bands that can react to anti-biotin mAb but not to anti-p53 mAb, which can bind to avidin coated plate and thus interfere with the assay. Although biotinylated p53-BCCP protein can be selectively immobilized from crude cell lysate directly onto the avidin coated microplates, without having to go through the purification steps, the obtained strong background and the compromised sensitivity of the antigen to differentiate weakly positive serum from those that were negative suggested that this system may not be suitable for preparing antigens to detect autoantibodies in human serum. However, it is still of interest to investigate whether using a smaller part of BCCP, as has been recently reported [17], to enable biotinylation of the tumor antigen or using strepavidin instead of avidin will improve its reactivity.

In contrast, directly immobilized (His)6-tagged p53 protein onto Ni^2+^-coated microplates followed by extensive washing with a series of buffers containing various concentrations of imidazole, was pure enough to differentiate the p53 autoantibody positive sera from those that were negative. In addition, the measured reactivity highly correlated with those obtained from using the purified (His)6-tagged p53 as antigen. The washing was comparable to those performed on nickel chelate affinity chromatography in order to purify (His)6-tagged p53; however, the immobilized antigens could be directly subjected to ELISA without having to go through a dialysing process. Moreover, the Ni^2+^-coated microplate is reusable, although there was a tendency towards an increase in optical density (data not shown); therefore, this would not be recommended for a clinical test.

One of the potential problems of utilizing (His)6-tagged p53 antigen directly from crude lysate to detected autoantibody is ensuring reproducibility of the assay when a new lot of bacterial cell lysate has been prepared. In this study, we tried to ensure reproducibility of the assay by culturing and preparing cell lysate exactly the same way as the previous one and the new lot of cell lysate was subjected to react with various concentrations of the commercial anti-p53 mAb (DO7) and some positive sera in order to titrate the optimal concentration of the new cell lysate to attain the same reactivity.

When reactivity of the directly immobilized (His)6-tagged p53 antigen from crude lysate was assayed with sera from lung cancer patients and healthy volunteers by an ELISA using a cut off based on the mean OD_450 nm _of sera from healthy volunteers with 3SD, 12 out of 68 cancer patients were positive. However, among these 12 patients only 9 patients (OD_450 nm _above 0.800) gave a 53 Kda reactive band when assessed by Western blot analysis. This discrepancy may be due to several possibilities. First, it is possible that the denatured conformation of p53 protein antigen presented in the Western blot analysis destroied some recognizable epitopes that were previously presented when the ELISA was performed. A second possibility may be that the cut-off value was based on only 22 healthy volunteers with a younger age and no history of smoking thus may under-represent the non-cancer condition. Therefore, a larger group of non-cancer volunteers needs to be investigated in order to decide a definitive cut-off value.

Numerous studies have demonstrated that cancer sera contained antibodies [18-20], which react with a unique group of autologous intracellular antigens known as TAAs. Although the mechanism leading to this immune response is not clearly understood, the target antigens are often cellular protein whose aberrant regulation and function could be linked to malignancy. The oncogenic nature of most TAAs has led the hypothesis that tumor associated autoantibodies are immunological reporters indicating aberrant cellular function associated with tumourigenesis. Many investigators have been interested in the use of autoantibodies as serological markers for cancer diagnosis [9,13,21], especially due to the general absence of these autoantibodies in non-cancer conditions. However, enthusiasm of this approach has been tempered by a low sensitivity. It has been demonstrated recently that the sensitivity of autoantibody detection in cancer patients was enhanced when using a panel of tumor-associated antigens instead of just one tumor antigen [22-25]. A simplified approach of developing a tumor antigen specific ELISA to detect autoantibodies in cancer patients reported in this study might thus enable a larger panel of TAAs to be investigated. Although detection of serum autoantibodies are shifting from ELISA platforms to microarray slide formats, microarray technology requires sophisticated fluorescence detection scanners they are not currently accessible in most parts of the world. Therefore, we believed the detection of serum autoantibodies by an ELISA platform will still be widely used for years to come, particularly in resource limited settings.

## Conclusion

This study has demonstrated a simplified approach of coating directly and specifically a tumor-associated antigen onto a microtiter plate. Although only one TAA, p53, was investigated in this study, our preliminary study showed that directly coated p53 antigen was able to identify p53 specific autoantibody producing serum and its reactivity highly correlated with those obtained using the purified p53 antigen.

## Methods

### Patient sera

Sixty-eight serum samples were collected from patients with non-small cell lung carcinoma (36 with adenocarcinomas and 32 with squamous cell carcinomas) admitted to the Maharaj Nakorn Chiang Mai hospital during 2005–2006. Out of these 68 cancer patients, 47 were smokers. For healthy volunteer controls, serum was collected from 22 blood donors with no known diagnosis of cancer and no history of smoking at the blood-banking department of Maharaj Nakorn Chiang Mai hospital. Mean (SD) age was 59.2 (9.91) and 26.4 (7.72) years in the cases and controls, respectively. All patients and blood donor volunteers gave informed consent prior to the collection of blood sample and the samples were stored at -70°C until analysed. The study was reviewed and approved by the research ethics committee of the faculty of Medicine, Chiang Mai University, Chiang Mai, Thailand.

### Vectors construction and recombinant proteins expression

Wild type full-length p53 cDNA (gifted from Dr John Lunec, Northern Institute for Cancer Research, University of Newcastle upon Tyne, UK) was cloned into BCCP (biotin carboxyl carrier protein) containing expression vector, SpT5.10/pAK400cB (a gift from Dr. Ville Santala, Department of Biotechnology, University of Turku, Finland) or hexahistidine-tag (His)6 containing vector, pET-15b(+) (purchased from Novagen, USA). The primer adapter sequences used for cloning of p53 cDNA into SpT5.10/pAK400cB vector through *NdeI *and *EcoRI *restriction sites were 5'-GAG GAG GAG GTC ATA TGG AGG AGC CGC AGT CAG AT-3' and 5'-GAG GAG GAG CTG GAT CCT TAG TCT GAG TCA GGC CCT TC-3' and primers used for cloning into pET-15b(+) vector through *NdeI *and *BamHI *restriction sites were 5'-GAG GAG GAG GTC ATA TGG AGG AGC CGC AGT CAG AT-3' and 5-'GAG GAG GAG CTG AAT TCG TCT GAG TCA GGC CCT TC'-3, respectively. Both of the established DNA constructs were verified by nucleotide sequencing.

To produce the recombinant proteins, SpT5.10/pAK400cB vector and pET15b(+) vector harbouring p53 encoding cDNA was transformed into *E.coli *Origami B cells (Novagen, USA) and *E.coli *strain BL21(DE3)pLyS (Novagen, USA), respectively. A fresh colony was inoculated into 5 ml SB culture medium (30 g/L tryptone, 15 g/L yeast extract, and 10 g/LMOPS, pH 7.0) supplement with 50 μg/ml chloramphenicol for bacteria carrying SpT5.10/pAK400cB vector or 100 μg/ml ampicillin for pET15b(+) vector and cultured at 37°C until OD_600 nm _reached 0.8, after which they were kept at 4°C overnight. The following morning, old culture media was discarded before resuspending bacterial cell pellet into 250 ml of fresh media. After obtaining 0.8 OD_600 nm_, expression of the recombinant proteins was induced with 1 mM isopropyl-β-D-thiogalactopyranoside (IPTG) and the cultured was continued at 37°C until harvesting at 3 hours after induction. In the case of SpT5.10/pAK400cB vector expressing in *E.coli *Origami B, during induction culture media was additionally supplemented with 4 μM of biotin (Sigma Aldrich, USA).

### Confirmation of p53 recombinant protein expression by Western blot analysis

Western blotting was performed to investigate the successful expression of p53 recombinant protein. For (His)6-p53 fusion protein expression, twenty micrograms of bacterial cell lysate from *Escherichia coli *strain BL21(DE3)pLyS expressing (His)6-p53 fusion protein was resolved on a 10% SDS polyacrylamide gels under reducing conditions and electrotransferred onto a PVDF membrane (PALL Gelman Laboratory, U.S.A). The same strain of bacteria transformed with pET15b(+) empty vector and cultured in the same way was also analysed as a negative control. The membrane was blocked with 5% non-fat milk in TBS, containing 0.05% Tween-20 (TBS-Tween) for 1 hour before being incubated with monoclonal antibodies specific for p53 (DO7, dilution 1:1000, Novacastra, USA) and histidine (H-3, dilution 1:1000, Santa Cruz Biotechnology, USA) for 1 hour at room temperature (RT), and with horseradish peroxidase-conjugated goat anti-mouse IgG (Dako, U.S.A) for 1 hour at RT, respectively.

In the case of detecting biotinylated p53-BCCP fusion protein, the SpT5.10/pAK400cB harbouring CD147 [26] encoding DNA was used as a negative control. After bacterial cell lysate was resolved on a 10% SDS polyacrylamide gels under reducing conditions and electrotransferred onto a PVDF membrane, the membrane was subjected to react with monoclonal antibodies specific for biotin (Anti-biotin-HRP conjugated, 1:1000 dilution, Zymed, USA) or p53, and then with horseradish peroxidase-conjugated goat anti-mouse IgG, respectively. The membrane was then washed 4 times with TBS-Tween (15 minutes each), immunoreactive protein was visualized by a chemiluminescence-based (ECL) procedure. Dual protein markers (GE Healthcare, Sweden) which could be detect by ECL were used to determine the molecular weight of the recombinant proteins.

### Purification of (His)6-p53 recombinant protein

The (His)6-p53 fusion protein was purified by using metal-affinity chromatography. *Escherichia coli *strain BL21(DE3)pLyS expressing (His)6-p53 fusion protein were harvested, frozen at -20°C overnight and resuspended in 1X binding buffer (6 M urea, 5 mM imidazole, 0.5 mM NaCl, and 20 mM Tris-HCl, pH 7.9). Bacterial pellet from 250 ml culture were lyzed in 30 ml of 1X binding buffer. The lysate was vertexed and passed through automatic pipette several times until it become clear before being centrifuged at 10000 g, 4 °C for 15 minutes and the supernatant was diluted with water to 0.5× but adjusted imidazole and NaCl concentration to 5 mM and 0.5 mM, respectively. The diluted cell lysate (30 ml, 1 volume) was then applied onto a HisTrap™FF crude (1 ml, GE Healthcare, Sweden). Unbound proteins were washed out with 2 volumes of binding buffer followed by 2 volumes of each washing buffer (0.5 M NaCl, 20 mM Tris-HCl, 6 M urea, pH 7.9) containing increasing concentration of imidazole (20 mM, 40 mM, 60 mM and 80 mM). The (His)6-p53 fusion protein was then eluted with 5 ml of 1 M imidazole. The eluted protein was dialysed against 3 changes of 25-folds volume excess of PBS pH 7.4 in regenerated cellulose tubular membrane (Cellu. Sep T2, MWCO: 6,000–8,000, Membrane Filtration Products, Inc., Texas, USA). 250 ml of bacterial cell culture with 100 mg total protein gave around 300 μg of the purified (His)6-p53 fusion proteins. Purified p53 fusion protein was further characterized by SDS-PAGE and coommassie blue staining.

### Examination of total proteins loaded on polyacrylamide gels by coommassie blue staining

The polyacrylamide gel carrying separated proteins was submerged into generous amount of coomassie blue staining solution (0.025% Coommassie Brilliant Blue R250 (GE Healthcare, Sweden), 40% (v/v) methanol, 7% (v/v) acetic acid) and incubate at room temperature for overnight. Next morning, the stained gel was de-stained by replacing coommassie blue staining solution with the de-stained solution I (40% (v/v) methanol, 7% '(v/v) acetic acid), and shaked slowly for 30 min. This removed the buck of the excess stain. The de-stained solution I was replaced with de-stained solution II (7% (v/v) acetic acid, 5% (v/v) methanol) and the solution was periodically until the gel background was clear.

### Identification of p53-atuoantibody positive sera by Western blot analysis

Purified full-length (His)6-p53 fusion proteins were resolved on a 10% SDS polyacrylamide gels under reducing conditions and electrotransferred onto a PVDF membrane (PALL Gelman Laboratory, U.S.A) after which it was blocked with 5% non-fat milk in TBS containing 0.05% Tween-20 (TBS-Tween) for 1 hour before being cut into strips according to the lanes of the polyacrylamide gel. Each strip of the membrane, containing about 0.5 μg of the purified (His)6-p53 proteins, was then incubated with each patient's serum diluted 1:200 with PBS, pH 7.4, for 1 hour at room temperature followed by interaction with 1: 3000 dilution of polyclonal rabbit anti-human immunoglobulin horseradish peroxidase (HRP)-conjugated antibody (DakoCytomation, USA) for 1 hour as a secondary antibody. The immunoreactive protein was then visualized by a chemiluminescence (ECL)-based procedure.

### Preparation of Ni^2+^-NTA coated microtiter plates

The Ni^2+^-NTA coated microtiter plate was prepared as described previously [27], except an ordinary ELISA carboxylated polystyrene microplate (polystyrene high BIND microplate, Corning life science, USA) was used instead of a plate coated with maleic anhydride (Pierce, USA) as the later is 20 times more expensive. Briefly, a microtiter plate was coated with N,N-bis [carboxymethy]lysine (Sigma Aldrich, USA) overnight and washed 3 times with 0.05% Tween 20 before blocking with 3% BSA in 50 mM Tris-HCl, pH 7.5, 150 mM NaCl, 0.05% Tween 20. The coated plate was washed with a series of buffers including 1) 50 mM Tris-HCl, pH 7.5, 500 mM imidazole, 0.05% Tween 20 2) 0.05% Tween 20 3) 100 mM EDTA, pH 8.0 and 4) 0.05% Tween before charging with 10 mM NiSO_4 _for 20 minutes at room temperature. After washing with 0.05% Tween 20 and then with 50 mM Tris HCl, 500 mM NaCl, pH 7.5, the plate was then ready to be used to capture (His)6-containing proteins.

### ELISA

Purified (His)6-p53 fusion proteins were diluted in 50 mM carbonate buffer, pH 9.6 to a final concentration of 1 μg/ml and 100 μl were applied onto a polystyrene high BIND microplate (Corning life science, USA). The plate was incubated overnight at 4°C. Next morning, after discarding the coating solution and washing 3 times with PBS-Tween (10 mM phosphate buffer, 150 mM NaCl, 0.05% Tween 20), the plate was blocked with 5% non-fat milk dissolved in PBS-Tween. To selectively immobilize (His)6-p53 fusion proteins from crude cell lysate onto microplate without prior purification, the cell lysate at the concentration of 100 μg/ml prepared from *Escherichia coli *strain BL21(DE3)pLyS expressing (His)6-p53 fusion protein was diluted in 0.5× binding buffer (around 700 μl of the original crude lysate was diluted to 10 ml in 0.5× binding buffer) and incubated in a Ni^2+^-NTA coated microtiter plates overnight at 4°C. The following morning, microplate was washed 4 times with a series of washing buffer (0.5 M NaCl, 20 mM Tris-HCl, 6 M urea, pH 7.9) containing increasing concentrations of imidazole (20 mM, 40 mM, 60 mM and 80 mM). The coated plate was then blocked with 0.5% non-fat milk dissolved in PBS-Tween. In the case of p53-BCCP fusion proteins readily biotinylated during expressing in *E.coli *Origami B, they were selectively immobilized using an avidin coated polystyrene high BIND microplate. A 96-well microplate was coated with 100 μl of 5 μg/ml of avidin dissolved in 50 mM carbonate buffer, pH 9.6 and incubated overnight at 4°C. After discarding the coating solution and washing with PBS-Tween for 4 times, all of the wells were blocked with PBS-Tween containing 5% non-fat milk. The plate was then incubated for 1 hour with 100 μg/ml of the crude cell lysate from transformed *E.coli *Origami B diluted in PBS-Tween.

All p53 antigen coated microplate was subjected to react with sera from lung cancer patients and healthy volunteers diluted 1:200 in PBS. The antigen-antibody reaction was allowed to occur at room temperature for 1 hour, after which the plate was washed with PBS containing 0.05% Tween 20, and bound antibodies were detected using HRP-conjugated goat anti-human Ig (DakoCytomation, USA). After 4 washes with PBS-Tween, a TMB substrate solution (Zymed, USA) was added and the color development was allowed for 20 minutes before being stopped with 0.1 N HCl. The intensity of color was then measured at 450 nm using a microtiter plate reader.

## Authors' contributions

RC designed and sought funding for the study, initiated coordination and drafted the manuscript. SP performed DNA cloning experiment and ELISA. BC, NL and SS recruited cancer patients for the study and provided clinical information. CT participated in design of the study. WK sought funding and participated in design of the study. All authors read and approved the final manuscript.
